# Supplementary material for: From sequence to enzyme mechanism using multi-label machine learning
Source: BMC Bioinformatics. 2014 May 19;15:150. doi: 10.1186/1471-2105-15-150 (PMC4229970; doi:10.1186/1471-2105-15-150)
Supplement: Additional file 2 — Java code of ml2db. Additional file ml2db_code.tar.gz contains the Java source code to run the multi-label machine learning experiments and save the results to database. The code’s Javadoc is included. [file 1471-2105-15-150-S2.zip › additional file 2/ml2db/ml2db/doc/main/ml2db/al/database/AlDataSaver.html]

AlDataSaver


JavaScript is disabled on your browser.


- Overview
- Package
- Class
- Use
- Tree
- Deprecated
- Index
- Help

- Prev Class
- Next Class

- Frames
- No Frames

- All Classes

- Summary:
- Nested |
- Field |
- Constr |
- Method

- Detail:
- Field |
- Constr |
- Method


main.ml2db.al.database

## Class AlDataSaver

- java.lang.Object
- - uk.ac.standrews.utils.main.database.DbManaged
  - - main.ml2db.al.database.AlDataSaver

- ---

    

  ```
  public class AlDataSaver
  extends uk.ac.standrews.utils.main.database.DbManaged
  ```

  Class

  Version:
  :   12 Jul 2011

  Author:
  :   Luna De Ferrari ldf(at)st-andrews.ac.uk

- - ### Constructor Summary

    Constructors

    Constructor and Description

    `AlDataSaver(uk.ac.standrews.utils.main.database.DbManager dbManager,
    java.lang.String resultsDirectoryPath)`
  - ### Method Summary

    Methods

    | Modifier and Type | Method and Description |
    | --- | --- |
    | `TableRowSaver` | `getExperimentTableRowSaver()` |
    | `java.lang.String` | `getFileName()` |
    | `java.lang.String` | `getFilePath()` File to save the experiment data to (as backup) |
    | `TableRowSaver` | `getUtilityTableRowSaver()` |
    | `int` | `save(uk.ac.standrews.utils.main.database.TableRow tableRow, int experimentId)` |
    | `void` | `saveUtilityEntry(int experimentId, int stepId, java.lang.String instanceId, double utility)` |
    | `void` | `setFileName(java.lang.String tableName, int experimentId)` |

    - ### Methods inherited from class uk.ac.standrews.utils.main.database.DbManaged

      `getDbConnection, getDbManager, getDbReader`
    - ### Methods inherited from class java.lang.Object

      `equals, getClass, hashCode, notify, notifyAll, toString, wait, wait, wait`

- - ### Constructor Detail


    - #### AlDataSaver

      ```
      public AlDataSaver(uk.ac.standrews.utils.main.database.DbManager dbManager,
                 java.lang.String resultsDirectoryPath)
      ```
  - ### Method Detail


    - #### getExperimentTableRowSaver

      ```
      public TableRowSaver getExperimentTableRowSaver()
      ```


    - #### getFileName

      ```
      public java.lang.String getFileName()
      ```


    - #### getFilePath

      ```
      public java.lang.String getFilePath()
      ```

      File to save the experiment data to (as backup)

      Returns:


    - #### getUtilityTableRowSaver

      ```
      public TableRowSaver getUtilityTableRowSaver()
      ```


    - #### save

      ```
      public int save(uk.ac.standrews.utils.main.database.TableRow tableRow,
             int experimentId)
      ```


    - #### saveUtilityEntry

      ```
      public void saveUtilityEntry(int experimentId,
                          int stepId,
                          java.lang.String instanceId,
                          double utility)
      ```


    - #### setFileName

      ```
      public void setFileName(java.lang.String tableName,
                     int experimentId)
      ```


- Overview
- Package
- Class
- Use
- Tree
- Deprecated
- Index
- Help

- Prev Class
- Next Class

- Frames
- No Frames

- All Classes

- Summary:
- Nested |
- Field |
- Constr |
- Method

- Detail:
- Field |
- Constr |
- Method
